# Oral vancomycin induced flushing syndrome in a multiple myeloma patient: A case report and review of the literature

**DOI:** 10.1097/MD.0000000000040640

**Published:** 2024-11-22

**Authors:** Min Zeng, HeMei Wang, Huiying Qiu, JunWei Gao

**Affiliations:** a Department of Clinical Pharmacy, Shanghai General Hospital, School of Medicine, Shanghai Jiao Tong University, Shanghai, China; b Department of Pharmacy, The Affiliated Hospital, Southwest Medical University, Luzhou, China; c Department of Pharmacy, People’s Hospital of Rizhao, Rizhao, China; d Department of Hematology, Shanghai General Hospital, School of Medicine, Shanghai Jiao Tong University, Shanghai, China.

**Keywords:** case report, Clostridium difficile colitis, multiple myeloma, oral vancomycin, Red man syndrome, vancomycin flushing syndrome

## Abstract

**Background:**

Patients with hematological malignancies are at high-risk of Clostridium difficile infection (CDI). Oral vancomycin is a first-line treatment for CDI. Vancomycin has been widely reported to induce flushing syndrome (also known as Red man syndrome), a well-known hypersensitivity reaction mostly occurs after intravenous administration. However, a few cases of flushing syndrome due to oral vancomycin have been reported.

**Methods:**

We reported a case of the 68-year-old male with Multiple Myeloma contracted suspected CDI during chemotherapy, oral vancomycin (125 mg po q6h) was initiated for CDI. Approximately 24 hours after receiving oral vancomycin, the patient developed vancomycin flushing syndrome with facial flushing and an erythematous rash on the abdomen and back, despite normal vancomycin duration and renal function (no obvious risk factors).

**Results:**

The patient was diagnosed with Oral vancomycin induced flushing syndrome. The symptoms resolved after withdrawal of vancomycin and 4 days of treatment with loratadine.

**Conclusion:**

Oral vancomycin-induced flushing syndrome is a rare complication that can occur in patients with CDI despite the absence of obvious risk factors. The underlying mechanism of oral vacomycin-induced flushing syndrome may be direct activation of mast cells following mast cell degranulation and histamine release via the MRGPRX2 receptor. However, this is just speculation and there are insufficient data, particularly in vivo data, to draw any conclusions. For patients with risk factors such as gastrointestinal pathology and renal insufficiency, monitoring of vancomycin serum concentration, mast cell degranulation, histamine release, and MRGPRX2 levels is recommended to avoid vancomycin flushing Syndrome, and vancomycin can still be used under supervision.

## 1. Introduction

Clostridium difficile infection (CDI) is one of the leading infection in patients with hematological malignancies.^[1,2]^ The incidence of CDI in adults with malignancy is 7% to 14%,^[3]^ while in children with hematological malignancies the incidence is between 11.1% to 21.0%.^[4]^ Antibiotics usage is the main cause of CDI as antibiotics provoke a intestinal microbial disorder allowing the colonization of Clostridium difficile.^[2]^ Other risk factors for CDI include weakened immune system, advanced age (≥65 y), CDI history, and recently hospitalized.^[2,5]^ Patients undergoing chemotherapy are at high-risk of CDI.^[2,6]^ Because patients receiving chemotherapy (being immunocompromised) often received broad-spectrum antibiotics, since bacterial infections are a major complication.^[2]^ Oral vancomycin is a mainstay treatment for CDI.^[7]^ However, vancomycin has been widely reported to induce flushing syndrome (also known as Red man syndrome), a well-known hypersensitivity reaction mostly occurs after intravenous administration with symptoms varying from flushing, erythematous rash, pruritus, mild to profound hypotension, and even cardiac arrest.^[8,9]^ Vancomycin flushing syndrome is a non-immune-mediated release of histamine from mast cells and basophils^[10]^ with an incidence between 4% and 50%.^[11]^

However, a few cases of flushing syndrome due to oral vancomycin have been reported. Here, we report a case of a 68-year-old male patient with multiple myeloma who developed symptoms compatible with flushing syndrome while receiving oral vancomycin for suspected CDI.

## 2. Case presentation

A 68-year-old male patient with multiple myeloma (for 1 month) was admitted to a hematology ward of Shanghai General Hospital (China) on August 7, 2023, for further treatment. He had a history of chronic obstructive pulmonary disease for over 30 years (Budesonide, Glycopyrronium Bromide and Formoterol Fumarate Inhalation Aerosol 2 puffs bid, and long-term home oxygen therapy), hypertension (extremely high risk) for about 20 years (amlodipine 5 mg qd), and cerebral infarction for 2 years (rosuvastatin 10 mg qd).

Prior to admission (28 days ago), the patient had a primary chemotherapy with Bortezomib and dexamethasone, during when he had a respiratory tract infection and showed improvement after receiving cefoperazone-sulbactam. Ten days before admission, the patient experienced recurrent cough with yellow, watery sputum, accompanied by fever with a maximum temperature of 38.2°C. After taking ibuprofen, his temperature was normal, but he developed scattered rashes all over his body which suspected to be adverse reaction of ibuprofen. Seven days before admission, the patient had a urinary catheter inserted, with a self-reported daily urine output of 800 to 1000 mL, occasionally with blood streaks and clot. Three days before admission, he experienced swelling and pain in both lower limbs, along with shortness of breath that was relieved by rest and oxygen therapy.

On admission, he presented with multiple dark red macules and papules on the trunk and limbs, and diagnosed with allergic dermatitis which was suspected as a adverse reaction of ibuprofen by the dermatology department. Ibuprofen was withdrawal immediately, meanwhile, oral loratadine (10 mg qd) and Calamine Lotion (15 mL applied topically tid) were initiated. The bone marrow smear showed active proliferation of the granulocytic and erythroid series, with platelet aggregation in the megakaryocytic lineage. So the patient was treated with ixazomib (4 mg po qw) for maintenance chemotherapy on August 7, 2023. About 5:00 pm of the same day, he developed a fever (39.4°C) with chills, and the chest CT revealed pulmonary inflammation. Ibuprofen (20 mg po prn) for antipyretic treatment and cefoperazone-sulbactam sodium (3 g ivgttt q8h) for empirical anti-infection therapy were started promptly. Two days later (on August 9, 2023), the patient’s fever persisted at 39.1°C and he showed a cloudy drainage urine. His C-reactive protein (CRP) and procalcitonin (PCT) were 166.3 mg/L and 34.25 ng/mL separately, with serum creatinine 114.7 µmol/L. Thus his anti-infection treatment was adjusted to meropenem (1 g ivgtt q8h) and teicoplanin (400 mg ivgtt qd). For the moment, the patient also reported a numbness in lower extremities which considered an adverse reaction caused by isatuximab. Symptomatic treatment with mecobalamin and vitamin B1 was initiated immediately. On August 11, 2023, the patient’s body temperature was within the normal range at 37.0°C. Additionally, his CRP had decreased to 109.0 mg/L and PCT was at 16.66 ng/mL, indicating that the current anti-infection treatment was effective. And his serum creatinine had decreased to 54.2 µmol/L. On August 17, 2023, the patient once again presented with a fever (38.2°C), a white blood cell count of 5.47 × 10^9^/L, a neutrophil count of 3.39 × 10^12^/L, and a creatinine of 52.2 µmol/L. Meanwhile, the patient reported watery stools 7 to 8 times per day without visible mucus or blood. Due to the patient’s medical history and ongoing antibiotic treatment, the possibility of pseudomembranous colitis couldn’t be excluded. Therefore, the patient received oral vancomycin (125 mg po qid) for the suspected CDI promptly. After administered vancomyin for about 24 hours (on August 18, 2023), the patient developed flushing on the face and erythematous rash on both abdomen and back. His blood pressure was 107/70 mmHg, heart rate at 80 beats per minute, and with normal body temperature at 36.5°C. Vancomycin flushing syndrome induced by oral vancomycin was considered, leading to the discontinuation of oral vancomycin, loratadine for symptomatic treatment, and a switch to teicoplanin (400 mg po bid) for CDI. Meanwhile, the chest CT showed slight absorption of pneumonia and meropenem had been prescribed for more than 1 week, a switch to cefoperazone-sulbactam sodium was made. On August 21, 2023, his vancomycin flushing syndrome (the rash on face, abdomen and back) had improved slightly, however, diarrhea persisted, and a bacterial culture of stool revealed Enterococcus faecalis positive. His PCT was 0.774 ng/mL. Tigecycline (50 mg ivgtt q12h) was added for anti-infection treatment, while cefoperazone-sulbactam sodium (3 g ivgtt q8h) and oral teicoplanin (200 mg po bid, for CDI) were continued. On August 23, 2023, his vancomycin flushing syndrome showed constant improvement and his diarrhea improved. Additionally, there also was an improvement in the patient’s CRP (14.4 mg/L) and PCT (0.257 ng/mL). Then oral teicoplanin was discontinued. On August 25, 2023, the patient only had scattered rashes on the abdomen and back, his body temperature was back to normal, the pneumonia was absorbed, and there was no more diarrhea. Then tigecycline was discontinued. On August 28, 2023, the patient discharged and was advised to continue oral loratadine (10 mg po qd) for the scattered rashes on the abdomen and back.

## 3. Discussion

In this case, we reported a male with Multiple Myeloma who developed the flushing syndrome about 24 hours after the treatment of oral vancomycin for CDI. Oral vancomyin is the first-line treatment for CDI.^[7]^ However, vancomycin may cause adverse reactions, including hypersensitivity reactions, kidney injury, and ototoxicity.^[12]^ Hypersensitivity reactions can manifest as anaphylaxis, vancomycin flushing syndrome, and linear IgA bullous dermatitis.^[8]^ Among these, vancomycin flushing syndrome is a rare anaphylactoid reaction^[13,14]^ that characterized by pruritus, flushing, erythematous rash, urticaria, hypotension, and even cardiac arrest.^[8]^

Usually, vancomycin flushing syndrome is associated with rapid intravenous administration of vancomycin, there have been even rare reports of flushing syndrome associated with oral, intraperitoneal, enteral, and local intra-wound application of vancomycin.^[8,9,11,13,15–22]^ Upon literature review, cases of vancomycin-induced flushing syndrome via non-intravenous administration are summarized in Table 1. As shown in Table 1, vancomycin flushing syndrome caused by non-intravenous administration was reported in 12 cases, including 7 cases of oral vancomycin (90 mg to 250 mg q6h),^[15–21]^ 2 cases of intraperitoneal vancomycin (1000–2000 mg),^[11,22]^ 1 case of vancomycin enteral (125 mg q6h),^[8]^ 1 case of vancomycin-loaded bone cement (1000 mg)^[13]^ and 1 case of local intra-wound vancomycin powder (500 mg).^[9]^ Patients with non-intravenous induced vancomycin flushing syndrome were predominantly elderly, with 66.67% (8/12) being over the age of 60, and the age ranged from 23 months to 82 years. Besides, the patients were mainly female, with 8 out of 12 cases being female. Moreover, the diagnoses for patients with non-intravenous vancomycin flushing syndrome including CDI (8 of 12),^[8,15–21]^ peritonitis (2 of 12)^[11,22]^ and prevention of surgical site infections (2 of 12).^[9,13]^ Furthermore, as can be seen from Table 1, risk factors for patients with non-intravenous vancomycin flushing syndrome including renal dysfunction (6/12),^[11,17,18,20–22]^ neutropenia (1/12),^[16]^ previous episode of vancomycin (IV) flushing syndrome (1/12),^[19]^ immunosuppression (1/12),^[20]^ and no significant risk factors (3/12).^[8,9,13]^ The onset of vancomycin flushing syndrome ranged from 45 minutes to 8 days after administration. Notably, 5 out of 7 patients with oral vancomycin flushing syndrome had a slow onset (at least 2 days). The main symptoms of vancomycin flushing syndrome were erythema and pruritus of the face, neck, arms, chest and back, with edema and mild hypotension in 1 patient. As summarized in Table 1, there were 4 patients who had recurrence of vancomycin flushing syndrome,^[11,15,16,22]^ 2 of whom received oral vancomycin^[15,16]^ and 2 of whom received intraperitoneal vancomycin.^[11,22]^

**Table 1:** Reported cases of vancomycin flushing syndrome via non-intravenous administrations.

Reference	Age (yr/mo)	Sex	Administration route	Vancomycin dose and serum level	Reactions	Onset time of reactions	Recovery time	Treatment	Risk factor	Reintroduce vancomycin	Reoccurrence of reactions	Details of reoccurred reactions	Diagnosis	Complications
Killian et al 1991^[15]^	67 yr	F	Oral	500 mg q6h NA	Pruritus (intense): face, neck, arm Erythema: face, neck, chest, arms	50min (after first vancom-ycin dose)	30min	None	High vancomycin dose	Yes (500 mg q6h)	Yes	Onset time: 60 min Reactions: Pruritus (intense): face, scalp, arm Erythema: face, neck, thorax, arms Treatment: diphenhydramine: 25 mg iv Recovery time: 50 min Vancomycin serum level: 3.3 μg/mL (trough centrations, 90 min after the sixth vancomycin dose), 4.1 μg/mL (peak centrations, 90 min after the sixth vancomycin dose)	CDI after antibiotic therapy for bronchitis	Bronchitis
Bergeron and Boucher 1994^[16]^	23 mo	M	Oral	90mg q6h NA	Edema: bilateral palpebral edema Swelling: lower lip Hypotension: slightly Redness: neck	60min	few hours	None	Neutropenia	Yes (90 mg q6h)	Yes	Onset time: 60 min Reactions: Edema: bilateral palpebral edema; Swell: lower lip Treatment: None Recovery time: NA Vancomycin serum level: 28.7 μg/mL (1 h after oral vancomyin on March 22); 5.7 μg/mL (immediately before oral vancomycin on March 25), 8.6 μg/mL (1 h after oral vancomycin on March 25)	CDI	Down’s syndrome and acute myeloblastic leukemia
Bailey et al 2008^[17]^	82 yr	F	Oral	250 mg qd NA	Pruritic: chest, back, neck and thighs Erythematous rash: chest, back, neck and thighs	4 d	NA	Stop vancomycin Regular antihistamines	Chronic Kidney Disease (CKD) stage 2	No	NA	NA	CDI after co-amoxicl-av therapy for a urinary tract infection	Atrial fibrillation, ischemic heart disease, chronic kidney disease, hypertension, osteoarthritis, (6 yr ago) a right hemicolectomy for Dukes B colorectal carcinoma
Nallasivan et al 2009^[18]^	58 yr	F	Oral	NA NA	Erythematous urticarial rash: stomach wall (slowly progressed)	3 d	2 wk	Stop vancomycin Regular antihistamines Topical skin creams	Renal dysfunction	No	NA	NA	CDI after co-amoxicl-av and piperacillin therapy for postoperati-ve pneumonia	Elective knee replacement, diabetes,osteoarthritis, hypertension
Arroyo-Mercado et al 2019^[19]^	75 yr	F	Oral	250 mg q6h NA	Flushing, erythema, and pruritus of the face, neck and upper torso	2 d	24 h	Stop vancomycin Diphenhydramine	Previous episode of Vancomycin (IV) flushing syndrome	No	NA	NA	CDI after antibiotic therapy for pneumonia	Asthma, diabetes and breast cancer
Yadav et al 2022^[20]^	68 yr	M	Oral	125 mg q6h NA	Pruritus, erythematous and non-blanching rash: face, neck, upper extremities	8 d	Few days	Stop vancomycin Regular antihistamines	Renal dysfunction Immunoco-mpromised	No	NA	NA	CDI pseudomembranous colitis	Chronic kidney disease stage 4
Kapitza et al 2023^[21]^	79 yr	F	Oral	250 mg q6h NA	Exanthema: face, trunk, arms	2 d	Few days	Dimetindene	Renal dysfunction	No	NA	NA	CDI after antibiotic therapy for pneumonia	Pneumonia
Barron et al 2018^[8]^	66 yr	F	Enteral	125 mg q6h 0.1 μg/mL	Red macules, papules and patches: thighs, torso, and back.	4 d	3 d	Stop vancomycin Chlorpheniramine	NA	No	NA	NA	CDI after first generation cephalospor-in for cellulitis	Diabetes, chronically ventilated, end-stage neurodegenerative disease
Domis et al 2014^[22]^	11 yr	M	Intraperitoneal	1000 mg/L 38.8 μg/mL	Rash Itching Agitation	45 min	NA	Diphenhydramine: 25 mg po	End-stage renal disease	Yes (500 mg/L)	No	NA	Bacterial peritonitis	End-stage renal disease
Möhlmann et al 2024^[11]^	33 yr	F	Intraperitoneal	2000 mg/1.5L 54.8 μg/mL	Flushing, pruritic rash: face, neck, shoulders swelling: lips	75 min	4 h	Clemastine: 2 mg po prn Dexamethasone: 8mg po prn	Renal dysfunction	Yes (1000 mg/1.5 L→500 mg/1.5 L)	No	Vancomycin serum level: 33.6 μg/mL (2 h after vancomycin intraperitoneal administration)	Peritonitis	Graft failure after a prior kidney transplantation
Chen et al 2018^[13]^	74 yr	F	Loaded bone cement	1000 mg NA	Erythematous rash and pruritis: face, trunk, lower extremities Hypotension (47/34 mm Hg)	45 min	1.5 h	Diphenhydramine: 30 mg iv Hydrocortisone: 100 mg iv	NA	No	NA	NA	Prophylacti-c use of antibiotic loaded bone cement	Osteoarthritis, total knee replacement
Nagahama et al 2018^[9]^	73 yr	M	Local intra-wound application	500 mg NA	Erythematous segmental rash: chest, upper abdomen	Several days	1 wk	Symptomati-c treatment	NA	No	NA	NA	Prophylacti-c use of surgical site infections	Parkinson’s disease underwent 2-stage deep brain stimulation implantation surgeries

CDI = Clostridium difficile diarrhea, F = female, M = male, NA = not available.

However, in most reported cases (9/12) of non-intravenous vancomycin flushing syndrome, serum concentrations of vancomycin had not been obtained.^[9,13,15–21]^ Specifically, vancomycin serum concentrations were not reported in any cases of oral vancomycin-induced flushing syndrome.^[15–21]^ Three cases reported vancomycin concentrations: 1 case with enteral vancomycin had a concentration of 0.1 μg/mL,^[8]^ while the other 2 cases with intraperitoneal vancomycin had concentrations of 38.8 μg/mL^[22]^ and 54.8 μg/mL,^[11]^ respectively. The limited data above suggested that vancomycin flushing syndrome may occur regardless of vancomycin concentration. Additionally, the cases of intraperitoneal vancomyscin indicated that gastrointestinal (GI) pathology may increase vancomycin absorption, leading to an increase in vancomycin serum concentration.

In general, vancomycin is considered to be poorly absorbed after oral or other non-intravenous administration, but has high intestinal concentrations after oral administration and has therefore become the mainstay of treatment for CDI.^[23]^ However, as the use of oral vancomycin has increased, so have concerns about the serum levels and toxicity of oral vancomycin. Risk factors for systemic vancomycin exposure from oral vancomycin in case studies may be highly variable and biased due to complex disease and publication bias. However, the study by Pettit et al added more comprehensive data on the absorption of oral vancomycin and risk factors. Risk factors associated with exposure to serum vancomycin (after oral administration) are high vancomycin dose (>500 mg/d), prolonged duration (>10 days), severe intestinal inflammation (or gastrointestinal [GI] pathology), renal insufficiency and intensive care unit (ICU) care.^[24]^

While Cimolai^[23]^ summarized 32 studies on the serum concentrations of oral and enteral vancomycin, the results outlined that vancomycin could be absorbed with a serum concentration ranging from 0 to 49.8 µg/mL, but the high concentration of vancomycin did not necessarily lead to vancomycin flushing syndrome. Similarly, Pettit et al^[24]^ only explored risk factors for vancomycin serum concentration after oral administration, but did not demonstrate an association between vancomycin concentration and vancomycin flushing syndrome.

Vancomycin Flushing Syndrome is a pseudo-allergic drug reaction that is independent of immunoglobulin E-dependent anaphylactic reactions.^[25]^ The underlying mechanism of vancomycin flushing syndrome has been attributed to the direct activation of mast cells following mast cell degranulation and histamine release after vancomycin challenge.^[25–27]^ Furthermore, the mast cell receptor Mas-Related G-Protein-coupled Receptor X2 (MRGPRX2) has been identified as a target of pseudo-allergic drug reactions by McNeil et al.^[28]^ However, vancomycin-induced mast cell degranulation and histamine release has only been studied in vitro with human cutaneous mast cells and rat peritoneal mast cells. Meanwhile, MRGPRX2 mediated vancomycin-stimulated mast cell degranulation and histamine release has also been demonstrated in vitro experiment.^[25–27]^ Currently, there is no sufficient data, particularly in vivo data, to suggest that vancomycin flushing syndrome is caused by mast cell degranulation and histamine release mediated by the mast cell receptor MRGPRX2.

As for non-intravenously induced vancomycin flushing syndrome, such as oral vancomycin-induced flushing syndrome, it has been hypothesized that risk factors such as the presence of gastrointestinal pathology (e.g. CDI, without intestinal integrity), renal dysfunction may facilitate the absorption of oral vancomycin, leading to a high vancomycin serum concentration, thus inducing vancomycin flushing syndrome. However, cases of vancomycin flushing syndrome have been documented despite low vancomycin serum concentrations (0.1 μg/mL).^[8]^ It turns out that vancomycin serum concentration doesn’t correlate vancomycin flushing syndrome.

Other possible explanation for non-intravenous vancomycin-induced flushing syndrome may be that both mast cells and the receptor MRGPRX2 are important in the pathogenesis of vancomycin flushing syndrome after non-intravenous (e.g. oral) administration, and that the local concentration of vancomycin or the amount of vancomycin that challenges mast cells also plays a role. In short, the patient with a high local vancomycin concentration and with sufficient mast cells and MRGPRX2 may experience vancomycin flushing regardless of vancomycin serum levels. Then the following reasons: MRGPRX2 is a low-affinity receptor and is heterogeneously expressed in different tissues or individuals, mast cells are heterogeneously distributed and the number of mast cells varies in different tissues or individuals, vancomycin serum levels can’t represent local vancomycin concentration, may explain why vancomycin serum concentration doesn’t correlate with vancomycin flushing syndrome. However, the above is just speculation. We don’t have enough data to draw conclusions about the underlying mechanism of oral vancomycin-induced flushing syndrome. Further research is needed to demonstrate the exact pathogenesis of vancomycin flushing syndrome after oral administration.

In this case, the patient was diagnosed with multiple myeloma and was receiving oral vancomycin at a dose of 125 mg q6h (known as low or standard dose) for CDI, with normal treatment duration and renal function. Approximately 24 hours after starting vancomycin, the patient presented with facial flushing and an erythematous rash on the abdomen and back, consistent with vancomycin-induced flushing syndrome. According to the Naranjo Adverse Drug Reaction Probability Scale,^[29]^ the rash was a probable reaction to vancomycin (score 6/13). Adverse reactions to co-administration of drugs such as isatuximab and meropenem were ruled out, as were discontinuation of vancomycin and response to symptomatic treatment despite ongoing administration of isatuximab and meropenem, providing evidence of vancomycin-induced flushing syndrome. Although vancomycin serum concentrations may be detectable in our patient, we haven’t obtained that data. Similar to the case reported by Bergeron and Boucher,^[16]^ the potential risk factors for the absorption of oral vancomycin in this patient are: The patient had a haematological tumor and was undergoing chemotherapy and CDI, which was vulnerable to gastrointestinal dysfunction, a complex clinical situation such as multiple complications and medications.

Although, based on available evidence, vancomycin serum concentration doesn’t correlate with vancomycin flushing syndrome. However, a detectable or high concentration after non-intravenous (e.g. oral) administration of vancomycin may indicate a high local vancomycin concentration and gastrointestinal pathology. In these circumstances, mast cells and the MRGPRX2 receptor may be activated, leading to vancomycin flushing. Therefore, for patients with risk factors, monitoring of vancomycin serum concentration, mast cell degranulation, histamine release, and MRGPRX2 levels are recommended, and vancomycin can still be used under supervision.

## 4. Conclusions

In summary, we present a case of a 68-year-old male with multiple myeloma who developed vancomycin flushing syndrome after receiving a normal dose of oral vancomycin (125 mg q6h) despite normal vancomycin duration and renal function. Vancomycin Flushing Syndrome is a pseudoallergic drug reaction. As with non-intravenously induced vancomycin flushing syndrome, the underlying mechanism may be direct activation of mast cells following mast cell degranulation and histamine release via the MRGPRX2 receptor. However, this is just speculation and we don’t have enough data to draw any conclusions. For patients with risk factors such as gastrointestinal pathology, monitoring of vancomycin serum concentration, mast cell degranulation, histamine release, and MRGPRX2 levels is recommended, and vancomycin can still be used under supervision.

## Acknowledgments

We acknowledge the support and contribution of the Department of Hematology, Shanghai General Hospital, School of Medicine, Shanghai Jiao Tong University, Shanghai, China, in providing expert medical counsel concerning the patient.

## Author contributions

**Conceptualization:** JunWei Gao.

**Data curation:** Min Zeng.

**Investigation:** Min Zeng, HeMei Wang.

**Resources:** Min Zeng, HeMei Wang.

**Writing – original draft:** Min Zeng.

**Writing – review & editing:** Huiying Qiu, JunWei Gao.
